# tRNA Function and Dysregulation in Cancer

**DOI:** 10.3389/fcell.2022.886642

**Published:** 2022-06-01

**Authors:** Tania Gupta, Mark G. Malkin, Suyun Huang

**Affiliations:** ^1^ Virginia Commonwealth University, Richmond, VA, United States; ^2^ Department of Neurology, School of Medicine, Virginia Commonwealth University, Richmond, VA, United States; ^3^ VCU Massey Cancer Center, Virginia Commonwealth University, Richmond, VA, United States; ^4^ Department of Human and Molecular Genetics, School of Medicine, Virginia Commonwealth University, Richmond, VA, United States; ^5^ Institute of Molecular Medicine, School of Medicine, Virginia Commonwealth University, Richmond, VA, United States

**Keywords:** tRNA, cancer, TRF, tiRNA, tumor, protein

## Abstract

Transfer RNA (tRNA) is a central component of protein synthesis and plays important roles in epigenetic regulation of gene expression in tumors. tRNAs are also involved in many cell processes including cell proliferation, cell signaling pathways and stress response, implicating a role in tumorigenesis and cancer progression. The complex role of tRNA in cell regulation implies that an understanding of tRNA function and dysregulation can be used to develop treatments for many cancers including breast cancer, colon cancer, and glioblastoma. Moreover, tRNA modifications including methylation are necessary for tRNA folding, stability, and function. In response to certain stress conditions, tRNAs can be cleaved in half to form tiRNAs, or even shorter tRNA fragments (tRF). tRNA structure and modifications, tiRNA induction of stress granule formation, and tRF regulation of gene expression through the repression of translation can all impact a cell’s fate. This review focuses on how these functions of tRNAs, tiRNA, and tRFs can lead to tumor development and progression. Further studies focusing on the specific pathways of tRNA regulation could help identify tRNA biomarkers and therapeutic targets, which might prevent and treat cancers.

## Introduction

Cancer continues to be one of the leading causes of death in the world, accounting for 13% of all deaths (Zhao & Li, 2021). Cancer cells are characterized by rapid cell growth, which must be supported through the reprogramming of metabolic pathways. Unlike healthy cells which primarily use oxidative phosphorylation for energy production, tumor cells primarily rely on anabolic pathways including aerobic glycolysis, fatty acid synthesis, and the pentose phosphate pathway to absorb nutrients that can be used to build macromolecules in order to meet the demands of the rapidly proliferating cell (Zhao & Li, 2021). Because approximately 70% of cell dry weight is protein, cancer cells especially depend upon high levels of protein production (Dolfi et al., 2013). In fact, as the cell proliferation rate increases, so does the protein synthesis rate per cell volume (Dolfi et al., 2013).

Protein production begins when genetically coded, hardwired DNA is first transcribed into messenger RNA (Yu et al., 2021) in the nucleus, which is then transported into the cytoplasm to be translated into protein that can be used by the cell. In the cytoplasm, tRNA translates each three-base codon on the mRNA into an amino acid. These amino acids form a chain known as a polypeptide, which can be processed into a protein. Because of the critical role that tRNAs play in protein production and cell survival, tRNA transcripts are tightly controlled before being post transcriptionally modified (Schaffer et al., 2019). These modifications are important for tRNA structure and function (Torres et al., 2014). The dysregulation of tRNAs and tRNA modifying enzymes has been implicated in a multitude of disorders such as neurodevelopmental disorders (Schaffer et al., 2019), type 2 diabetes, and the development and proliferation of many cancers including breast, bladder, and colorectal cancer (Torres et al., 2014). This is not surprising considering that tRNA synthesis is managed by many oncogenes and tumor suppressors (Huang et al., 2018). The understanding that we have regarding the role of tRNA in cancer cells suggests that there is at least a correlation, if not a causal relationship, between tRNA malfunction and cancer cell proliferation. This review will concentrate primarily on the association between tRNA methylation, tRNA fragments, and selenoproteins and cancer development.

## tRNA Structure and Functions

**FIGURE 1:**
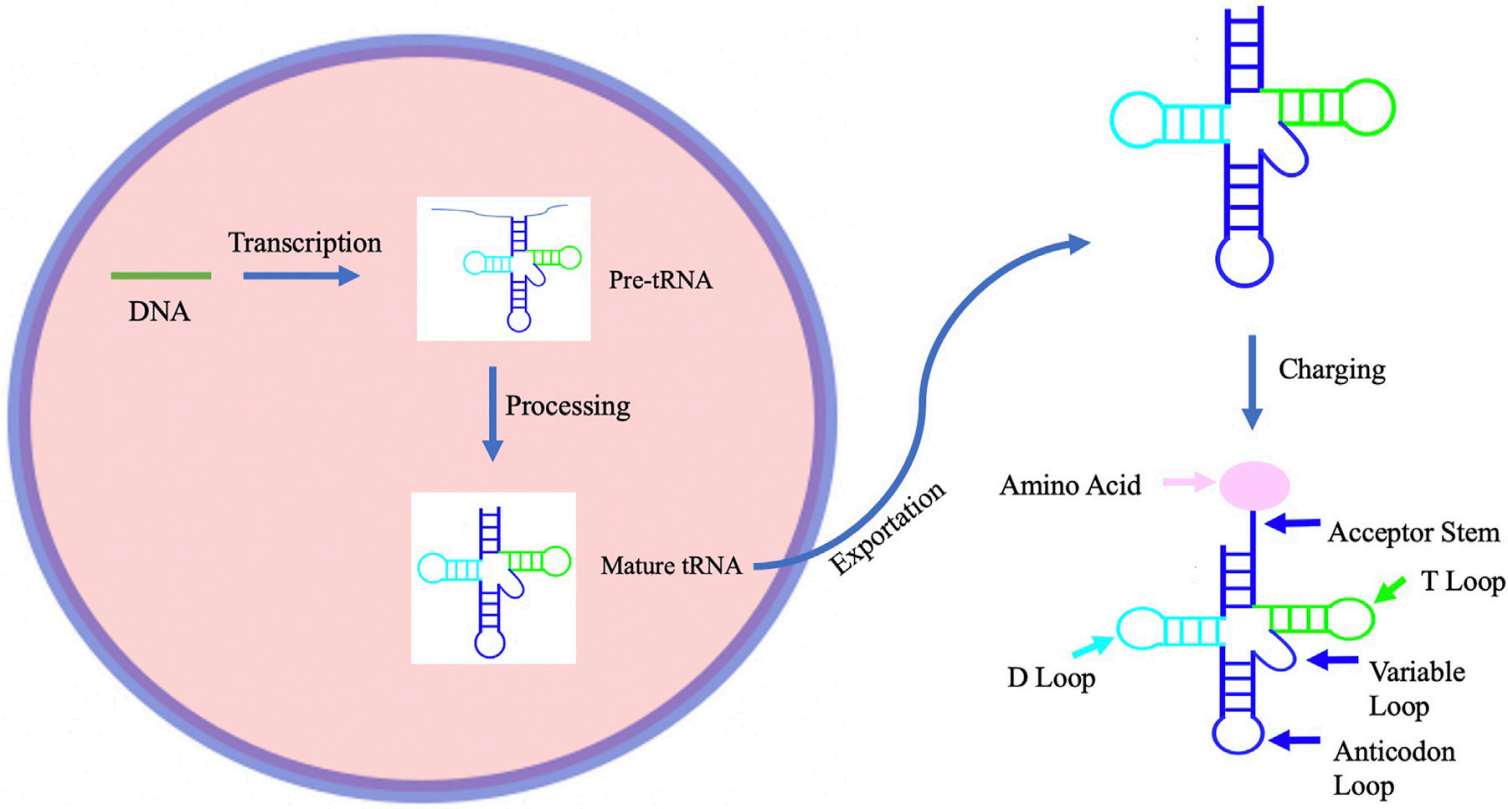
A schematic representation of the process of tRNA biogenesis. tRNA biogenesis begins with transcription of tRNA to form pre-tRNA. This pre-tRNA is then processed to form a mature tRNA before being exported from the nucleus. The mature tRNA is then ready to be charged with an amino acid by an aminoacyltRNA synthetase.

tRNA structure and modifications play a dominant role in its function. During tRNA synthesis, tRNA precursors must be transcribed within the nucleus, before being modified and exported out of the nucleus (Figure 1). In eukaryotes, this process begins with RNA polymerase III and transcription factors TFIIIB and TFIIIC transcribing tRNA genes into pre-tRNAs (Santos et al., 2019). Post-transcription, tRNAs are processed to form mature tRNA. During procession, RNase P removes the 5′leader of the pre-tRNA transcript, La protein binds to the U tract of the 3′ end, and Rnase Z cleaves the discriminator nucleotide (Berg and Brandl). The basic structure of most tRNA molecules includes an acceptor stem and D-arm, which work together to recognize aminoacyl tRNA synthetase; the anticodon arm, which ensures that the correct amino acid is added to the peptide chain; the T-arm, which aides in ribosome interaction; and the variable loop (Lyons et al., 2018). However, despite this general structure, the positions of the anticodon and acceptor stems may differ among tRNAs, indicating a flexibility that is necessary for tRNA interaction with many different molecules within the cell (Kuhn, 2016). Upon development of the mature tRNA, modifying enzymes may then add modifications (Santos et al., 2019). Ninety-three tRNA modifications have been identified (Berg & Brandl, 2021), with tRNAs undergoing the most modifications of any RNA species at a median of thirteen modifications per tRNA in eukaryotes (Pan, 2018). Although the primary function of most tRNA modifications is to either stabilize the tertiary structure of the tRNA or influence the codon-anticodon recognition (Lyons et al., 2018), each modification has a different effect on tRNA function. Furthermore, the role of tRNA within the cell extends beyond the translation of mRNA. tRNA has been shown to be involved in many other cell processes including cell signaling pathways and the cellular response to stress.

## tRNA Modifications in Cancer Cells

tRNA modifications are epigenetic and can adjust the rate of translation to meet the cell’s needs (Hou et al., 2015). Post-transcriptional modifications such as hydroxylation, acetylation, and deamination (Suzuki, 2021) can contradict the tRNA’s default mode of reading codons using Watson-Crick base pairing rules through impacting the accuracy of translation, the efficiency of translation, or the abundance of certain tRNA species (Endres et al., 2019). For example, the N6-threonylcarbamoyladenosine (t^6^A) tRNA modification, which is formed through addition of a Thr residue to the N^6^ position of adenine aides in codon recognition, aminoacylation, and translocation, while queuosine, a hypermodified guanosine derivative, at position 34 can impact the rate of translation elongation (Suzuki, 2021). Additionally, tRNA methyltransferase ALKBH8 may impact mRNA translation through catalyzing the hydroxylation of cm^5^U or mcm^5^U into chm^5^U or mchm^5^U, respectively, in tRNA^Gly^ (U*CC) (Fu et al., 2010). This function may be linked to cancer cell progression as ALKBH8 has been found to be upregulated in bladder cancer and increase ROS production in cancer cells (Fu et al., 2010). Some hypomodified tRNAs are degraded (Santos et al., 2019) by exonucleases and removed through a process known as rapid tRNA decay. However, some hypomodified tRNAs remain, indicating that tRNA modifications are not static and are dependent on cellular conditions (Suzuki, 2021). For example, although the transfer RNA guanine transglycosylase completely post transcriptionally modifies specific tRNAs to exchange guanine for queuine in terminally differentiated somatic cells, it incompletely modifies undifferentiated rapidly growing cells (Pathak et al., 2005).

Because of the abnormal cellular conditions in cancer cells, tRNA modifications are especially different. Due to the cancer cell’s rapid proliferation rate, blood supply is often not enough to sustain the cancer cell, leading the cell to reach a state of hypoxia, or low oxygenation. This can cause oxidative stress. Oxidative stress can activate a multitude of tumor-activating signaling cascades, some of which may upregulate tRNA modifying enzymes. These modifying enzymes will catalyze tRNA modification, thus increasing translation of target tRNA molecules (Endres et al., 2019). This is supported by findings that anticodon wobble uridine tRNA modifications are upregulated in breast, bladder, and melanoma cancer cells (Hawer et al., 2018). Although the downstream effects of these modifications differed based on the cancer cell type, U34 modifications were shown to support the shift in translation in cancer cells and promote cancer cell growth (Hawer et al., 2018). Conversely, a decrease in wobble uridine modifications was demonstrated to be detrimental in hematopoiesis. In a mouse model, an inactivation of Elp3, the catalytic subunit of the elongator that modifies wobble uridine in tRNAs, led to p53 mutation-driven leukemia/lymphoma (Rosu et al., 2021). These functions of tRNA modification are further complicated by debate over whether these tRNA modifications directly impact the expression of stress-related proteins or act in stress signaling (Kirchner & Ignatova, 2014).

**FIGURE 2:**
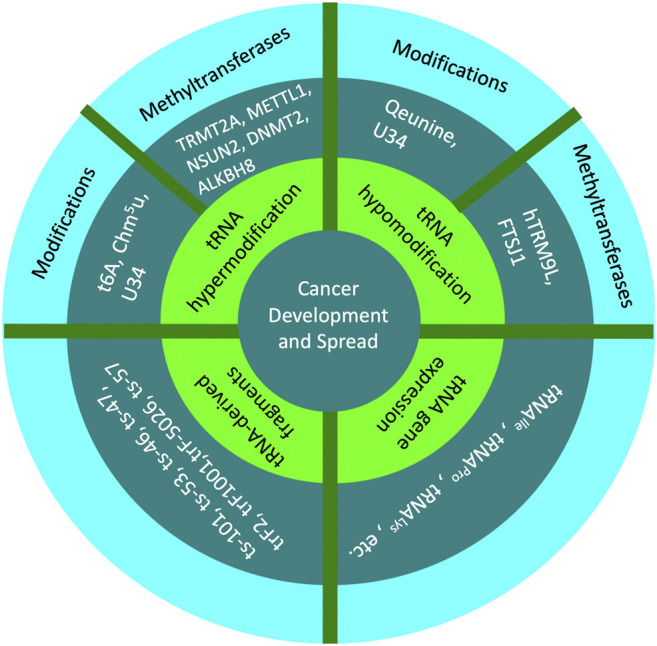
This diagram lists examples of tRNA hypermodification, tRNA hypomodification, tRNA gene expression, and tRNA-derived fragments that can lead to cancer development and spread. For tRNA hypermodification and tRNA hypomodification, the diagram includes tRNA modifications and tRNA methyltransferases associated with cancer development and spread.

Dysregulation of some of these modifications can have serious consequences for protein synthesis and have been linked to certain cancers and genetic disorders (Lin et al., 2018) (Figure 2). For instance, hypomodification of tRNA by the transfer RNA guanine transglycosylase has been found to be associated with Dalton’s Lymphoma ascites, lung cancer, ovarian cancer, and human leukemia (Pathak et al., 2005). Additionally, because of the importance of functioning protein synthesis for synapse development (Kirchner & Ignatova, 2014), neuronal cells may be particularly vulnerable to tRNA dysregulations.

Even the steps prior to tRNA modification can regulate gene expression. Aminoacyl-tRNA synthetase (Kim et al., 2012), a ligase which catalyzes the tRNA esterification to its cognate amino acid, has been shown to have other domains that are unrelated to this primary function, but can form complexes that are linked to cancers including glioblastoma (Kim et al., 2012). These interactions amongst ARSs may significantly impact the phenotype of glioblastoma, and thus influence the long-term survival of patients with glioblastoma (Kim et al., 2012). Aminoacylases such as Leucyl-tRNA synthetase and methionyl-tRNA synthetase, which charge tRNAs with leucine and methionine respectively, are associated with tumor formation or cell death (Rubio Gomez and Ibba, 2020). Additionally, misacylation by ARSs beyond the normal rate can cause changes in gene expression and may lead to cancer development, as these mistranslation errors can lead to polypeptide chains with unpredictable issues (Santos et al., 2019). These abnormal interactions of ARSs within the cell can change the prognosis of glioblastoma patients (Kim et al., 2012). These findings demonstrate the major effects of ineffective or mis-regulated tRNA modification on cell biology.

## tRNA Methylation and Cancer

The quantity and frequency of tRNA modifications varies, depending on factors such as chemical imbalances (Kimura et al., 2020) and cell cycle stage (Patil et al., 2012). A depletion of certain tRNA modifications can impact the rate of translation of cell cycle genes which are specifically coded by tRNAs (Lin et al., 2018). Many modifications play an active role in the cell’s stress response in both healthy and diseased cells. One such modification, tRNA methylation is conducted by tRNA methyltransferases (Endres et al., 2019) and is necessary for tRNA folding, stability, and function (Lin et al., 2018). tRNA methylation is also an important modulator of cell proliferation and differentiation. Low tRNA methylation has been shown to decrease the global translation rate (Papatriantafyllou, 2012).

Although in healthy cells tRNA modifications can help prevent disease, changes in the rate of translation of cell cycle genes can disrupt cell cycle regulation and lead to tumorigenesis (Figure 2). In such cells, oxidative stress can activate the mitogen-activated protein kinase cascade (Li et al., 2021), which targets the human tRNA methyltransferase 9 like (*hTRM9L*) gene in order to suppress cell growth (Endres et al., 2019). The upregulation of *hTRM9L* has been shown to use different pathways to express tumor suppressive qualities in colon, lung, and ovarian cancers (Endres et al., 2019). However, a loss of the *hTRM9L* region on chromosome 8 has been found in breast, bladder, prostate, and colon cancer, and the hTMRM9L enzyme has been shown to downregulate the oncogene and cell cycle regulator, cyclin D1 (Endres et al., 2019). In lung cancer tissues, *hTRM9L* downregulation was also shown be associated with poor prognosis (Bian et al., 2021). Although *hTRM9L* has been shown to have different methods of tumor suppression of each of these cancers, it had a universal effect of reducing tumorigenesis (Da Ros et al., 2018).

The tRNA methyltransferase, FTSJ1, can mediate 2′O methylation of tRNA (He et al., 2020). It has been shown that FTSJ1 have a tumor-suppressor effect in healthy cells, but was downregulated in non-small cell lung cancers (Wang et al., 2022) (Bian et al., 2021). This downregulation of FTSJ1 resulted in fewer tRNA modifications, particularly of 2′-O-methyladenosine (Luchman et al.) modification, and an increase in tumor cell proliferation (Bian et al., 2021). In contrast, tRNA methyltransferase 1 (METTL1), which mediates the formation of N^7^-methylguanine (m^7^G) modification (Ying et al., 2021), has been shown to promote tumor cell proliferation and increase chemoresistance (Li et al., 2021). METTL1 levels were found to not only be higher in cancer patients, but also have an inverse relationship with survival for cancers such as bladder cancer (Li et al., 2021). Because METTL1 levels increased with increasing glioma grade, METTL1 expression levels may be able to be used to predict glioma prognosis (Li et al., 2021). METTL1 was also shown to promote hepatocarcinoma, lung cancer, and intrahepatic cholangiocarcinoma proliferation through catalyzing m^7^G tRNA modification (Chen et al., 2021; Dai et al., 2021; Ma et al., 2021). Furthermore, depletion of METTL1 led to decreased m^7^G tRNA modifications and the overall global translation rate, suggesting that METTL1 may be able to be used as an anti-cancer target (Orellana et al., 2021). High levels of METTL1 can also promote chemoresistance to cisplatin and docetaxel in advanced nasopharyngeal carcinoma through increasing m^7^G tRNA modification and upregulating the WNT/β-catenin signaling pathway (Chen et al., 2022).

Additionally, the m^c^5 tRNA methyltransferases NSUN2 and DNMT2, which also play a role in translation regulation, have been found to be overexpressed in many cancer types including oral, colon and breast cancer cells, respectively (Endres et al., 2019; Dong & Cui, 2020). The NSUN2 and METTL1 tRNA modification genes have been associated with resistance to anti-cancer therapy (Hawer et al., 2018). However, deletion of NSUN2 and METTL1 in HeLa cells increased sensitivity to the anti-cancer drug 5-fluorouracil (Suzuki, 2021). Previously mentioned, ALKBH8, can produce mcm^5^ U tRNA modifications, has been shown to be upregulated in the event of DNA damage (Patil et al., 2012). Mcm^5^ U levels have been shown to oscillate throughout the cell cycle and help regulate its progression (Patil et al., 2012). Mcm^5^ U modifications have been shown to slow cell cycle progression in *S. cerevisiae*, and a similar result would be expected in humans in response to ALKBH8 upregulation (Patil et al., 2012). In contrast, ALKBH1, a member of the same AlkB family as ALKBH8 catalyzes the demethylation of N1 -methyladenosine (m^1^A) in tRNAs in response to glucose availability (Liu et al., 2016). This decreases the use of tRNA in protein synthesis and the overall translation rate (Liu et al., 2016). The effects of ALKBH1 can differ based on cancer type. Although the upregulation of AlkBH1 was found to promote proliferation of gastric cancer cells, it was found to correlate to better survival in pancreatic cancer and lung cancer patients (Wang et al., 2022).

TRMT2A, another tRNA methyltransferase, is highly expressed in HER2+ breast cancer cells and may indicate a higher chance of relapse (Suzuki, 2021). Other tRNA modification genes such as TRIT1, TRMT12, and ELP1 are associated with lung cancer, breast cancer, and melanoma, respectively (Hawer et al., 2018). Although many modifications are upregulated in cancer cells, tRNA hypomodification is also often found in these diseased cells (Suzuki, 2021). Deficiencies of wybutosine, a nucleoside important for codon recognition and reading frame maintenance found on eukaryotic tRNA^Phe^, has been detected in hepatoma, neuroblastoma, and colorectal cancer (Suzuki, 2021). This modification induces frame shifting and leads to nonsense-mediated RNA decay (Suzuki, 2021).

## Effects of tRNA Gene Expression on Cancer Cell Regulation

Understanding tRNA gene expression may also provide insight regarding cell regulation. Close to half of the tRNA genes within the human population are constitutively silent or poorly expressed. These genes may have extra-transcriptional functions, such as acting as insulators, which can block gene expression (Torres, 2019). In another form of tRNA variation, tRNAs known as isodecoders, are associated with the same anticodon, yet differ elsewhere on the tRNA body (Czech et al., 2010). These tRNA isodecoders vary in their efficiency of translational suppression despite having similar aminoacylation levels (Geslain & Pan, 2011), suggesting that some isodecoders may play a larger role in the regulation of gene expression (Czech et al., 2010). Evidence for the function of isodecoders in gene regulation can be seen in the differing translational efficiency rates of isodecoders despite their association with the same codon (Geslain & Pan, 2010). Specifically, Leu and Ser derived tRNA isodecoders have been shown to have varying levels of stop-codon suppression efficiencies, while all tRNA^Ala^ have demonstrated low suppression activity (Geslain & Pan, 2010). Even the roles of aminoacyl tRNA synthetase extend beyond protein production. Some aminoacyl tRNA synthetases such as seryl tRNA synthetase have been shown to be associated with metabolism. In healthy cells, glucose mediates the acetylation of seryl tRNA synthetase, causing it to translocate into the nucleus and suppress *de novo* lipid biosynthesis. However, breast cancers inhibit this translocation leading to abnormal lipid biosynthesis (Zhao et al., 2021). These findings exhibit the complexity and variation within the roles of tRNA in the cell. Although the roles of silent tRNA and tRNA isodecoders are still being discovered, their ability to affect gene regulation suggests that misfunction can have detrimental effects on gene expression. tRNA gene expression most likely plays an important role in cell proliferation in both healthy and cancerous cells. Although the exact mechanism of tRNA regulation is unknown, the key role that tRNA regulation plays in cell proliferation suggests that the upregulation of tRNA can also induce the development of tumorigenesis.

As tumor cells rapidly proliferate, they require the necessary cellular components to maintain a high growth rate. Although most likely more of an effect than a cause of cancer proliferation, numerous tumor cells have been shown to have elevated levels of tRNAs (Gingold et al., 2014). For example, breast cancer cells have been shown to upregulate some tRNAs by up to ten times (Pavon-Eternod et al., 2009). Oncogenes play an important role in meeting the high level of gene transcription at the mRNA level. Through obstructing tumor suppressors ability to inhibit RNA polymerase III transcription in healthy cells, oncogenes can increase Pol III transcription, thus increasing tRNA transcription (Bian et al., 2021). Oncogenes are also able to selectively induce the expression of certain tRNAs and repress the expression of other tRNAs in order to aid in this rapid rate of cell proliferation (Santos et al., 2019). Specifically, the initiator methionine tRNA has been shown to be induced the most in proliferating cells (Pavon-Eternod et al., 2013), while the selenocysteine tRNA has been shown to be repressed in many cancerous and proliferating cells (Barroso et al., 2014) (Luchman et al., 2014) (Hudson et al., 2012). These induced tRNAs have been shown to prefer codons enriched in proliferation genes (Gingold et al., 2014). tRNA synthesis in glioblastoma has also been shown to be linked to *de novo* GTP biosynthesis caused by increased *Impdh2* expression (Kofuji et al., 2019). This upregulation of *Impdh2* has also been shown to be positively correlated with increased glioma malignancy and negatively correlated with patient survival (Kofuji et al., 2019). Similarly, high levels of tRNA^Ile^, tRNA^Pro^, tRNA^Lys^ have been shown to be related to tumor differentiation in lung adenocarcinoma tissues and paracarcinoma tissues (Bian et al., 2021) (Figure 2). This tRNA upregulation may be linked not only to tumorigenesis, but may also to cancer patient prognosis, suggesting that it may be used as a marker of cancer development (Santos et al., 2019).

Conversely, the downregulation of tRNA can be used to limit cell growth and proliferation. Targeting certain tRNA genes that are necessary for mRNA translation will lead to cell cycle exit and decreased protein translation. This will decrease cell proliferation, acting as a tumor suppressant (Yang et al., 2020). One such gene is SOX4, a transcription factor that controls the expression of some tRNA genes. Although depending on the type of cancer SOX4 can act as a tumor suppressor or an oncogene, it has been shown to limit glioblastoma cell proliferation (Zhang et al., 2014). Through binding to certain tRNA genes, SOX4 can repress the expression of tRNAs and thus reduce protein synthesis (Yang et al., 2020). In fact, high SOX4 expression was found to be associated with better prognosis for glioblastoma patients (Zhang et al., 2014). Other pathways that decrease the rate of protein translation could be used to limit the spread of cancers. The drug Norcantharidin (Zhang et al., 2015) has demonstrated the capability to slow protein translation through targeting microRNA to treat cancer (Zhang et al., 2015). Norcantharidin has been used to treat certain malignant cancers and has been shown to suppress invasion by glioblastoma cells (Zhang et al., 2015). The success of Norcantharidin suggests the possibility that other drugs that reduce the rate of protein translation may be used as treatment (Zhang et al., 2015). Leucyl-tRNA synthetase, an aminoacyl tRNA synthetase that charges tRNA (Leu) with L-leucine, may also be targeted as an anticancer treatment partially due to its overexpression in cancer cells and association with the p21 protein, which may act as a tumor suppressor (Gao et al., 2015).

## tRNA-Derived Fragments and Cancer Development

During times of stress, the cell has many measures in place to prevent long-term damage to the cell and conserve energy. Since protein translation has a high energy and resource demand, the cell takes measures to reduce protein production when resources are scarce. tRNA is often implicated in these measures due to its pivotal role in protein translation. Before protein translation, aminoacylation attaches an amino acid to a tRNA, thus charging it. However, under nutritional stress, aminoacylation levels of tRNAs within the cell can change (Raina & Ibba, 2014) and uncharged tRNAs can act as signaling molecules of cellular processes (Nunes et al., 2020). For example, uncharged tRNAs may activate the general amino acid control pathway during times of cellular stress (Zaborske et al., 2009). This pathway induces protein kinase Gcn2p to phosphorylate eukaryotic initiation factor-2 (Zaborske et al., 2009), a necessary factor for the start of translation, thus reducing its activity (Zaborske et al., 2009). Protein translation can also be rapidly inhibited through the cleavage of the 3′CCA terminal sequence of tRNAs or the reuptake of cytoplasmic tRNAs into the nucleus during times of nutritional stress (Kirchner & Ignatova, 2014).

In mammalian cells under nutritional, biological, physicochemical, and oxidative stress conditions, tRNA’s may be cut into fragments known as tRNA-derived small RNAs (tsRNAs). TsRNAs include half-tRNA molecules, called tiRNAs (Zhu et al., 2019), which are cleaved by the nuclease angiogenin (ANG), and are about 30–35 nucleotides in length (Yamasaki et al., 2009) (Ivanov et al., 2011). Certain tiRNAs have the ability to reduce protein production, induce stress granule formation, and interfere with siRNA-mediated silencing of stress-response genes in mammalian cells (Kirchner & Ignatova, 2014). Even shorter than tiRNAs molecules, are tRNA fragments (tRF) (Sobala & Hutvagner, 2013), which are between 13 and 20 nucleotides in length and are derived from the cleavage of mature or pre-tRNA by ANG (Raina & Ibba, 2014). There are five types of tRFs including tRF-1, tRF-2, tRF-3, tRF-5, and i-tRF (Zhang et al., 2020). Most tRFs are either induced in response to cellular stress of constitutively expressed (Lyons et al., 2018). Specific tRF functions include RNA silencing, translation regulation, and epigenetic regulation (Yu et al., 2020).

While tRFs make up only a fraction of the tRNA pool (Raina & Ibba, 2014), they can still have a major impact on the cell’s survival and proliferation. For example, 3′U and 5′ tRFs can be found in very high levels in actively proliferating cells (Sobala & Hutvagner, 2013). Additionally, in response to stress conditions such as starvation, oxidative stress, and heat shock (Lyons et al., 2018), tRF levels tend to be upregulated. For example, sodium arsenite stress, has been shown to lead to the demethylation of tRNAs, which can make them more prone to cleavage by ANG, leading to the generation of tRFs (Yu et al., 2021). These tRFs can induce translation repression and stress-granule assembly (Sobala & Hutvagner, 2013). In certain cases, tRFs are able to inhibit protein translation by preventing peptide bond formation (Sobala, 2013) or acting as signal transducers (Czech et al., 2010). In almost all cancers, ANG expression is increased, potentially in order to increase tRF expression and, in turn, cancer proliferation (Zeng et al., 2020).

tRNA-derived fragments play a multitude of roles in both cancer development and suppression (Figure 2). tRFs have been shown to reduce cell progression through limiting kinase activity, impacting mRNA stability, regulating reverse transcription, and regulating apoptosis (Zhu et al., 2019). Some tRNA-derived fragments are known to be associated with certain cancers. For example, ts-101, ts-53, ts-46, and ts-47 have been found to be downregulated in lung cancer (Yu et al., 2020). Additionally, colon adenomas have been shown to downregulate ts-53 and ts-101, and chronic lymphocytic leukemia cancer cells have been shown to downregulate ts-46, ts-47, ts-49, ts-53, and ts-101 (Zeng et al., 2020). tRF-1001, which is derived from tRNA^Ser^, has also been shown to promote cancer cell proliferation (Lyons et al., 2018). The upregulation of tRF-Leu-CAG in non-small cell lung cancers (NSCLCs) has also been shown to increase cell proliferation through upregulating the activity of aurora kinase A, an important kinase in mitosis (Bian et al., 2021). Mutations of tRNA fragments may also to cancer cell proliferation, such as ts-53 and ts-101 mutations found in chronic lymphocytic leukemia and lung cancer (Zeng et al., 2020).

tRFs may influence cancer progression through regulation of gene expression during cellular stress. During cellular stress, tRFs associate with RNA binding proteins that would otherwise bind oncogene transcripts and increase cell proliferation. This has been shown to hold true for the RNA binding protein Y-box binding protein (YBX1) in several cancers (Zhu et al., 2019). Specifically, in breast cancer cells, tRF-2s from tRNA^Glu^, tRNA^Asp^, tRNA^Gly^ and tRNA^Tyr^ have been shown to suppress metastasis through binding to YBX1, thus inhibiting its engagement with oncogenic mRNAs (Zeng et al., 2020). High YBX1 levels have been associated with poor prognosis of breast cancer patients and relapse following surgical resection (Shibata et al., 2018). YBX1 levels have also been directly correlated with poor prognosis for patients suffering from ovarian and prostate cancer (Shibata et al., 2018). Additionally, 5′-tRFs have been found to promote proliferation and migration of high-grade serous ovarian carcinoma cells through downregulation of HMBOX1 (Hu et al., 2022).

As another response to stress conditions, such as a change in cell pH or decrease in mitochondrial transmembrane potential, the cell may undergo apoptosis. Apoptosis is important for cell survival, as it can prevent the uncontrolled growth characteristic of cancer cells. tRNA may play a major role in this stress response process and the regulation of apoptosis. Apoptosis occurs through the release of cytochrome c from the mitochondria, which activates caspase-9 in the Apaf-1 apoptosome. Caspase-9 can then activate caspase-3, which will execute apoptosis. tRNA has been found to be able to directly inhibit apoptosis through scavenging for cytochrome c inside and outside the mitochondria (van Raam & Salvesen, 2010) and through repressing cytochrome c by binding to it (Huang et al., 2018). This suppression of cytochrome c prevents apoptosis and may lead to tumor proliferation (Mei et al., 2010). Further understanding of tRNA’s anti-apoptotic function may be useful for cancer therapies (Mei et al., 2010). Such therapies may target these tRNAs in order to induce apoptosis and prevent cancer cell proliferation.

tRNA fragments may also be used as biomarkers for cancers. tRFs derived from tRNA^Phe(GAA)^ and tRNA^Lys(CUU)^ may act as biomarkers of progression-free disease survival in prostate tumors (Endres et al., 2019). Other tRNA fragments may be used as biomarkers for cancers, such as 5′-tiRNAs which have shown promise as potential noninvasive biomarkers for breast cancer and head and neck squamous cell carcinoma (Zeng et al., 2020). A better understanding of tRF biomarkers may help elucidate possible biomarkers. For example, tRF-5026a was shown to regulate PTEN/PI3/AKT signaling pathway and decrease gastric cancer cell proliferation, suggesting that tRF-5026 may be used as a biomarker for gastric cancer (Zhu et al., 2021).

Because of the strong association between cancer proliferation and tRFs, tRF regulation could be used to develop cancer treatment. The ability of tRF regulation to prevent cancer progression has been demonstrated in lung cancer cells, where the overexpression of tRNA signatures ts-46 and ts-47 inhibited further growth and survival of two lung cancer cell lines (Yu et al., 2020). Additionally, tRFs have been identified as a therapeutic target in the treatment of hepatocellular carcinoma after blockage of tRF-3^LeuCAG^, a tRNA fragment important for ribosome biogenesis, led to tumor cell apoptosis (Yu et al., 2021).

Conversely, tRFs and tiRNAs may impact cancer cell resistance to chemotherapeutics. For example, tRNA-derived fragments tDR-0009 and tDR-7336 were shown to be upregulated and increased chemoresistance of doxorubicin in triple-negative breast cancer (Zeng et al., 2020). Ts-57s and ts-46s were also found to be related to breast cancer chemoresistance to lapatinib (Zeng et al., 2020). tRNA-derived fragments may also increase chemoresistance through inhibition of the eukaryotic translation inhibiting factor 4 g (Zhu et al., 2019), which can block expression of the adenosine triphosphate-binding cassette (ABC) transporter. This transporter is important for effluxing anti-cancer drugs across cell membranes (Zhang et al., 2020). Additionally, tRFs and tiRNAs produce stress granules, which have been shown to make glioblastoma cells resistant to the anticancer drug bortezomib (Zhang et al., 2020). YBX1, as previously discussed, may also play a role in the drug resistance of breast cancer cells through promoting antiestrogen resistance, and decreasing the effectiveness of endocrine therapeutic drugs for estrogen receptor positive (ER-positive) breast cancer patients (Shibata et al., 2018). tiRNA-5s have also been shown to lead to phospho-eIF2ɑ-independent stress granule assembly, which has been associated with chemotherapeutic resistance (Zeng et al., 2020).

## Selenoprotein Expression and Cancer Development

Selenium (Se) is an essential micronutrient that has been demonstrated to have many positive health benefits, including potentially preventing cancer cell differentiation (Murdolo et al., 2017). Selenoproteins (SEPs), which contain selenium, can differentially impact cancer development through antioxidant activity (Short & Williams, 2017). Selenoprotein development begins when dietary selenium in the form of selenomethionine undergoes metabolism in the liver to produce selenocysteine, which can then be degraded by selenocysteine lyase to yield selenide. These selenoproteins are formed when specialized tRNA translate the UGA site of mRNA as selenocysteine, rather than recognizing it as a termination signal (Jameson & Diamond, 2004). The tRNA is able to recognize this site due to the presence of the Sec insertion sequence (Jameson & Diamond, 2004) in the 3’ untranslated region of the mRNA (Jameson & Diamond, 2004).

Although Selenoproteins are widely recognized for their antioxidant activity, they also may impact angiogenesis, growth factor signaling, and the inhibition of apoptosis, which may either support or repress tumorigenesis (Short & Williams, 2017). Selenoprotein expression may also effect DNA stability and oncogene activation (Murdolo et al., 2017).

The biological mechanism SEPs use to exert their anticancer effects is uncertain. However, SEPs such as selenoprotein P (SELP), glutathione peroxidases (Jameson & Diamond, 2004), thioredoxin reductases (TXNRD) and selenoprotein F (SEP15) have been shown to be able to regulate tumorigenesis through impacting cancer-related signaling proteins (Jia et al., 2020). The effect of stress-related (selenoproteins) SEPs on tumorigenesis differs depending on the organ and cancer type. Glutathione peroxidase 4 (GPX4) provides cell protection from oxidative stress-induced cell death (Becker et al., 2014) and may be found in high levels in cancer cells, causing resistance to chemotherapeutics (Yang et al., 2014). GPX4 has been shown to regulate ferroptosis in large B cell lymphoma cells, renal cell carcinomas (Yang et al., 2014), and head and neck cancer cell lines (Shin et al., 2018). Inhibition of transcription factor Nrf2 and silencing of p62 were found to sensitize head and neck cancer cells to GPX4 inhibitors, thus inducing ferroptosis and providing a potential treatment to overcome chemoresistance (Shin et al., 2018). Targeting GPX4 with dihydroartemisinin (Yi et al., 2020) treatment (Yi et al., 2020) was also shown to be successful in causing glioblastoma cell death through increasing cellular ROS levels and inducing ferroptosis (Yi et al., 2020). Single-nucleotide polymorphisms (SNPs) in selenoprotein genes such as SELNOP and GPX (Jia et al., 2020) may impact the efficiency of selenoprotein synthesis as well as the risk of disease. SNPs in selenoprotein S (SEPS) have been linked with lung, breast, prostate, colorectal, bladder, and thyroid cancers (Short & Williams, 2017).

Because selenoprotein expression is determined explicitly by the expression of Sec tRNA (Carlson et al., 2004), differing expression of Sec tRNA may be used to study the role of Se in cancer progression. For example, interbreeding of Sec tRNA transgenic mice with prostate cancer resulted in more high-grade lesions. However, Sec tRNA transgenic mice had no change in hepatocellular tumor number compared to wild type mice when crossed onto TGF-alpha transgenic background (Short & Williams, 2017).

Hypoxic conditions can reduce selenoprotein synthesis at the posttranscriptional level, through decreased Sec tRNA levels (Becker et al., 2014). The two isoforms of Sec tRNA are encoded on *Trsp* (Carlson et al., 2018), a single copy gene found in eukaryotes (Serrão et al., 2018). Deletion of the *Trsp* gene completely eliminates selenoprotein expression (Carlson et al., 2018) and was shown to increase oxidative stress and increase reactive oxygen species (ROS) accumulation in macrophages or the liver of mice with cancer (Serrão et al., 2018), while excision of *Trsp* in mammary glands led to increased mammary tumors and decreased survival (Serrão et al., 2018). Additionally, sec-tRNA^sec^ gene mutation or deletions have also been linked to cancers such breast, colon, and prostate cancer (Serrão et al., 2018).

## Discussion

tRNA structure, modification, upregulation, and downregulation not only change depending on cell type, but can also change in response to cell conditions and can impact cell proliferation. How and to what extent tRNAs are modified or cleaved can determine cell survival. The many functions of tRNA and its derivatives contribute to the complex role tRNAs play within healthy cells, which only becomes further complicated in tumor cells. The unique roles tRNA derivatives play in different cancers (Huang et al., 2018) combined with tRNAs’ secondary structure and chemical modifications, make tRNAs challenging to study (Schaffer et al., 2019). Because tRNA derivatives are often specific to certain cancers, they may be used as targets for chemotherapeutics or as biomarkers of disease. A more thorough understanding of how these aspects of tRNA impact tumor cell suppression, tumor cell activation, and treatment resistance can provide valuable information that is useful for the development of potential therapies for individuals diagnosed with cancer. However, barriers such as drug resistance and tumor heterogeneity continue to challenge the development of cancer treatments (Hu et al., 2022).

## References

[B1] Barroso M. Florindo C. Kalwa H. Silva Z. Turanov A. A. Carlson B. A. (2014). Inhibition of Cellular Methyltransferases Promotes Endothelial Cell Activation by Suppressing Glutathione Peroxidase 1 Protein Expression. J. Biol. Chem. 289 (22), 15350–15362. 10.1074/jbc.M114.549782 24719327 PMC4140892

[B2] Becker N.-P. Martitz J. Renko K. Stoedter M. Hybsier S. Cramer T. (2014). Hypoxia Reduces and Redirects Selenoprotein Biosynthesis. Metallomics 6 (5), 1079–1086. 10.1039/c4mt00004h 24700164

[B3] Berg M. D. Brandl C. J. (2021). Transfer RNAs: Diversity in Form and Function. RNA Biol. 18 (3), 316–339. 10.1080/15476286.2020.1809197 32900285 PMC7954030

[B4] Bian M. Huang S. Yu D. Zhou Z. (2021). tRNA Metabolism and Lung Cancer: Beyond Translation. Front. Mol. Biosci. 8, 659388. 10.3389/fmolb.2021.659388 34660690 PMC8516113

[B5] Carlson B. A. Lee B. J. Tsuji P. A. Copeland P. R. Schweizer U. Gladyshev V. N. (2018). Selenocysteine tRNA[Ser]Sec, the Central Component of Selenoprotein Biosynthesis: Isolation, Identification, Modification, and Sequencing. Methods Mol. Biol. 1661, 43–60. 10.1007/978-1-4939-7258-6_4 28917036 PMC5836751

[B6] Carlson B. A. Novoselov S. V. Kumaraswamy E. Lee B. J. Anver M. R. Gladyshev V. N. (2004). Specific Excision of the Selenocysteine tRNA[Ser]Sec (Trsp) Gene in Mouse Liver Demonstrates an Essential Role of Selenoproteins in Liver Function. J. Biol. Chem. 279 (9), 8011–8017. 10.1074/jbc.M310470200 14660662

[B7] Chen B. Jiang W. Huang Y. Zhang J. Yu P. Wu L. (2022). N7-methylguanosine tRNA Modification Promotes Tumorigenesis and Chemoresistance through WNT/β-catenin Pathway in Nasopharyngeal Carcinoma. Oncogene 41, 2239–2253. 10.1038/s41388-022-02250-9 35217794

[B8] Chen Z. Zhu W. Zhu S. Sun K. Liao J. Liu H. (2021). METTL1 Promotes Hepatocarcinogenesis via M 7 G tRNA Modification‐dependent Translation Control. Clin. Transl. Med 11 (12), e661. 10.1002/ctm2.661 34898034 PMC8666584

[B9] Czech A. Fedyunin I. Zhang G. Ignatova Z. (2010). Silent Mutations in Sight: Co-variations in tRNA Abundance as a Key to Unravel Consequences of Silent Mutations. Mol. Biosyst. 6 (10), 1767. 10.1039/c004796c 20617253

[B10] Da Ros M. De Gregorio V. Iorio A. Giunti L. Guidi M. de Martino M. (2018). Glioblastoma Chemoresistance: The Double Play by Microenvironment and Blood-Brain Barrier. Ijms 19 (10), 2879. 10.3390/ijms19102879 PMC6213072 30248992

[B11] Dai Z. Liu H. Liao J. Huang C. Ren X. Zhu W. (2021). N7-Methylguanosine tRNA Modification Enhances Oncogenic mRNA Translation and Promotes Intrahepatic Cholangiocarcinoma Progression. Mol. Cell 81 (16), 3339–3355. e3338. 10.1016/j.molcel.2021.07.003 34352206

[B12] Dolfi S. C. Chan L. L.-Y. Qiu J. Tedeschi P. M. Bertino J. R. Hirshfield K. M. (2013). The Metabolic Demands of Cancer Cells Are Coupled to Their Size and Protein Synthesis Rates. Cancer Metab. 1 (1), 20. 10.1186/2049-3002-1-20 24279929 PMC4178206

[B13] Dong Z. Cui H. (2020). The Emerging Roles of RNA Modifications in Glioblastoma. Cancers 12 (3), 736. 10.3390/cancers12030736 PMC7140112 32244981

[B14] Endres L. Fasullo M. Rose R. (2019). tRNA Modification and Cancer: Potential for Therapeutic Prevention and Intervention. Future Med. Chem. 11 (8), 885–900. 10.4155/fmc-2018-0404 30744422 PMC8356697

[B15] Fu Y. Dai Q. Zhang W. Ren J. Pan T. He C. (2010). The AlkB Domain of Mammalian ABH8 Catalyzes Hydroxylation of 5-methoxycarbonylmethyluridine at the Wobble Position of tRNA. Angew. Chem. Int. Ed. 49 (47), 8885–8888. 10.1002/anie.201001242 PMC3134247 20583019

[B16] Geslain R. Pan T. (2010). Functional Analysis of Human tRNA Isodecoders. J. Mol. Biol. 396 (3), 821–831. 10.1016/j.jmb.2009.12.018 20026070 PMC2822071

[B17] Geslain R. Pan T. (2011). tRNA: Vast Reservoir of RNA Molecules with Unexpected Regulatory Function. Proc. Natl. Acad. Sci. U.S.A. 108 (40), 16489–16490. 10.1073/pnas.1113715108 21933958 PMC3189024

[B18] Gingold H. Tehler D. Christoffersen N. R. Nielsen M. M. Asmar F. Kooistra S. M. (2014). A Dual Program for Translation Regulation in Cellular Proliferation and Differentiation. Cell 158 (6), 1281–1292. 10.1016/j.cell.2014.08.011 25215487

[B19] Hawer H. Hammermeister A. Ravichandran K. Glatt S. Schaffrath R. Klassen R. (2018). Roles of Elongator Dependent tRNA Modification Pathways in Neurodegeneration and Cancer. Genes 10 (1), 19. 10.3390/genes10010019 PMC6356722 30597914

[B20] He Q. Yang L. Gao K. Ding P. Chen Q. Xiong J. (2020). FTSJ1 Regulates tRNA 2ʹ-O-Methyladenosine Modification and Suppresses the Malignancy of NSCLC via Inhibiting DRAM1 Expression. Cell Death Dis. 11 (5), 348. 10.1038/s41419-020-2525-x 32393790 PMC7214438

[B21] Hou Y.-M. Gamper H. Yang W. (2015). Post-transcriptional Modifications to tRNA-A Response to the Genetic Code Degeneracy. Rna 21 (4), 642–644. 10.1261/rna.049825.115 25780173 PMC4371315

[B22] Hu Y. Cai A. Xu J. Feng W. Wu A. Liu R. (2022). An Emerging Role of the 5′ Termini of Mature tRNAs in Human Diseases: Current Situation and Prospects. Biochimica Biophysica Acta (BBA) - Mol. Basis Dis. 1868 (2), 166314. 10.1016/j.bbadis.2021.166314 34863896

[B23] Huang S.-Q. Sun B. Xiong Z.-P. Shu Y. Zhou H.-H. Zhang W. (2018). The Dysregulation of tRNAs and tRNA Derivatives in Cancer. J. Exp. Clin. Cancer Res. 37 (1), 101. 10.1186/s13046-018-0745-z 29743091 PMC5944149

[B24] Hudson T. S. Carlson B. A. Hoeneroff M. J. Young H. A. Sordillo L. Muller W. J. (2012). Selenoproteins Reduce Susceptibility to DMBA-Induced Mammary Carcinogenesis. Carcinogenesis 33 (6), 1225–1230. 10.1093/carcin/bgs129 22436612 PMC3514862

[B25] Ivanov P. Villen J. Gygi S. P. Anderson P. Anderson P. (2011). Angiogenin-induced tRNA Fragments Inhibit Translation Initiation. Mol. Cell 43 (4), 613–623. 10.1016/j.molcel.2011.06.022 21855800 PMC3160621

[B26] Jameson R. R. Diamond A. M. (2004). A Regulatory Role for Sec tRNA[Ser]Sec in Selenoprotein Synthesis. RNA 10 (7), 1142–1152. 10.1261/rna.7370104 15208449 PMC1370604

[B27] Jia Y. Dai J. Zeng Z. (2020). Potential Relationship between the Selenoproteome and Cancer. Mol. Clin. Oncol. 13 (6), 1. 10.3892/mco.2020.2153 33133596 PMC7590431

[B28] Kim Y.-W. Kwon C. Liu J.-L. Kim S. H. Kim S. (2012). Cancer Association Study of Aminoacyl-tRNA Synthetase Signaling Network in Glioblastoma. PloS one 7 (8), e40960. 10.1371/journal.pone.0040960 22952576 PMC3432027

[B29] Kimura S. Srisuknimit V. Waldor M. K. (2020). Probing the Diversity and Regulation of tRNA Modifications. Curr. Opin. Microbiol. 57, 41–48. 10.1016/j.mib.2020.06.005 32663792 PMC7722113

[B30] Kirchner S. Ignatova Z. (2014). Emerging Roles of tRNA in Adaptive Translation, Signalling Dynamics and Disease. Nat. Rev. Genet. 16 (2), 98–112. 10.1038/nrg3861 25534324

[B31] Kofuji S. Hirayama A. Eberhardt A. O. Kawaguchi R. Sugiura Y. Sampetrean O. (2019). IMP Dehydrogenase-2 Drives Aberrant Nucleolar Activity and Promotes Tumorigenesis in Glioblastoma. Nat. Cell Biol. 21 (8), 1003–1014. 10.1038/s41556-019-0363-9 31371825 PMC6686884

[B32] Kuhn C.-D. (2016). RNA Versatility Governs tRNA Function. BioEssays 38 (5), 465–473. 10.1002/bies.201500190 26990636

[B33] Li D. Gao G. Yao Y. Li K. Mashausi D. Li D. (2015). A Human Leucyl-tRNA Synthetase as an Anticancer Target. Ott 8, 2933–2942. 10.2147/OTT.S88873 PMC4610879 26508878

[B34] Li L. Yang Y. Wang Z. Xu C. Huang J. Li G. (2021). Prognostic Role of METTL1 in Glioma. Cancer Cell Int. 21 (1), 633. 10.1186/s12935-021-02346-4 34838021 PMC8627054

[B35] Lin S. Liu Q. Lelyveld V. S. Choe J. Szostak J. W. Gregory R. I. (2018). Mettl1/Wdr4-Mediated m7G tRNA Methylome Is Required for Normal mRNA Translation and Embryonic Stem Cell Self-Renewal and Differentiation. Mol. Cell 71 (2), 244–255. e245. 10.1016/j.molcel.2018.06.001 29983320 PMC6086580

[B36] Liu F. Clark W. Luo G. Wang X. Fu Y. Wei J. (2016). ALKBH1-Mediated tRNA Demethylation Regulates Translation. Cell 167 (7), 1897. 10.1016/j.cell.2016.11.045 27984735

[B37] Luchman H. A. Villemaire M. L. Bismar T. A. Carlson B. A. Jirik F. R. (2014). Prostate Epithelium-specific Deletion of the Selenocysteine tRNA Gene Trsp Leads to Early Onset Intraepithelial Neoplasia. Am. J. Pathology 184 (3), 871–877. 10.1016/j.ajpath.2013.11.025 PMC3936312 24447801

[B38] Lyons S. M. Fay M. M. Ivanov P. (2018). The Role of RNA Modifications in the Regulation of tRNA Cleavage. FEBS Lett. 592 (17), 2828–2844. 10.1002/1873-3468.13205 30058219 PMC6986807

[B39] Ma J. Han H. Huang Y. Yang C. Zheng S. Cai T. (2021). METTL1/WDR4-mediated m7G tRNA Modifications and m7G Codon Usage Promote mRNA Translation and Lung Cancer Progression. Mol. Ther. 29 (12), 3422–3435. 10.1016/j.ymthe.2021.08.005 34371184 PMC8636169

[B40] Mei Y. Stonestrom A. Hou Y.-M. Yang X. (2010). Apoptotic Regulation and tRNA. Protein Cell 1 (9), 795–801. 10.1007/s13238-010-0107-x 21113408 PMC2992325

[B41] Murdolo G. Bartolini D. Tortoioli C. Piroddi M. Torquato P. Galli F. (2017). “Selenium and Cancer Stem Cells,” in Selenium and Selenoproteins in Cancer. Editor Kenneth D. T. a. F. G. (Academic Press), 136, 235–257. 10.1016/bs.acr.2017.07.006 29054420

[B42] Nunes A. Ribeiro D. R. Marques M. Santos M. A. S. Ribeiro D. Soares A. R. (2020). Emerging Roles of tRNAs in RNA Virus Infections. Trends Biochem. Sci. 45 (9), 794–805. 10.1016/j.tibs.2020.05.007 32505636

[B43] Orellana E. A. Liu Q. Yankova E. Pirouz M. De Braekeleer E. Zhang W. (2021). METTL1-mediated m7G Modification of Arg-TCT tRNA Drives Oncogenic Transformation. Mol. Cell 81 (16), 3323–3338. 10.1016/j.molcel.2021.06.031 34352207 PMC8380730

[B44] Pan T. (2018). Modifications and Functional Genomics of Human Transfer RNA. Cell Res. 28 (4), 395–404. 10.1038/s41422-018-0013-y 29463900 PMC5939049

[B45] Papatriantafyllou M. (2012). tRNA Methylation Controls Translation Rate. Nat. Rev. Mol. Cell Biol. 13 (9), 540. 10.1038/nrm3424 22850818

[B46] Pathak C. Jaiswal Y. K. Vinayak M. (2005). Hypomodification of Transfer RNA in Cancer with Respect to Queuosine. RNA Biol. 2 (4), 143–148. 10.4161/rna.2.4.2417 17114931

[B47] Patil A. Dyavaiah M. Joseph F. Rooney J. P. Chan C. T. Y. Dedon P. C. (2012). Increased tRNA Modification and Gene-specific Codon Usage Regulate Cell Cycle Progression during the DNA Damage Response. Cell Cycle 11 (19), 3656–3665. 10.4161/cc.21919 22935709 PMC3478316

[B48] Pavon-Eternod M. Gomes S. Geslain R. Dai Q. Rosner M. R. Pan T. (2009). tRNA Over-expression in Breast Cancer and Functional Consequences. Nucleic Acids Res. 37 (21), 7268–7280. 10.1093/nar/gkp787 19783824 PMC2790902

[B49] Pavon-Eternod M. Gomes S. Rosner M. R. Pan T. (2013). Overexpression of Initiator Methionine tRNA Leads to Global Reprogramming of tRNA Expression and Increased Proliferation in Human Epithelial Cells. RNA 19 (4), 461–466. 10.1261/rna.037507.112 23431330 PMC3677255

[B50] Raina M. Ibba M. (2014). tRNAs as Regulators of Biological Processes. Front. Genet. 5, 171. 10.3389/fgene.2014.00171 24966867 PMC4052509

[B51] Rosu A. El Hachem N. Rapino F. Rouault-Pierre K. Jorssen J. Somja J. (2021). Loss of tRNA-Modifying Enzyme Elp3 Activates a P53-dependent Antitumor Checkpoint in Hematopoiesis. J. Exp. Med. 218 (3). 10.1084/jem.20200662 PMC7849823 33507234

[B52] Rubio Gomez M. A. Ibba M. (2020). Aminoacyl-tRNA Synthetases. RNA 26 (8), 910–936. 10.1261/rna.071720.119 32303649 PMC7373986

[B53] Santos M. Fidalgo A. Varanda A. S. Oliveira C. Santos M. A. S. (2019). tRNA Deregulation and its Consequences in Cancer. Trends Mol. Med. 25 (10), 853–865. 10.1016/j.molmed.2019.05.011 31248782

[B54] Schaffer A. E. Pinkard O. Coller J. M. (2019). tRNA Metabolism and Neurodevelopmental Disorders. Annu. Rev. Genom. Hum. Genet. 20, 359–387. 10.1146/annurev-genom-083118-015334 PMC6716996 31082281

[B55] Serrão V. H. B. Silva I. R. da Silva M. T. A. Scortecci J. F. de Freitas Fernandes A. Thiemann O. H. (2018). The Unique tRNASec and its Role in Selenocysteine Biosynthesis. Amino Acids 50 (9), 1145–1167. 10.1007/s00726-018-2595-6 29948343

[B56] Shibata T. Tokunaga E. Hattori S. Watari K. Murakami Y. Yamashita N. (2018). Y-box Binding Protein YBX1 and its Correlated Genes as Biomarkers for Poor Outcomes in Patients with Breast Cancer. Oncotarget 9 (98), 37216–37228. 10.18632/oncotarget.26469 30647855 PMC6324687

[B57] Shin D. Kim E. H. Lee J. Roh J.-L. (2018). Nrf2 Inhibition Reverses Resistance to GPX4 Inhibitor-Induced Ferroptosis in Head and Neck Cancer. Free Radic. Biol. Med. 129, 454–462. 10.1016/j.freeradbiomed.2018.10.426 30339884

[B58] Short S. P. Williams C. S. (2017). Selenoproteins in Tumorigenesis and Cancer Progression. Adv. Cancer Res. 136, 49–83. 10.1016/bs.acr.2017.08.002 29054422 PMC5819884

[B59] Sobala A. Hutvagner G. (2013). Small RNAs Derived from the 5′ End of tRNA Can Inhibit Protein Translation in Human Cells. RNA Biol. 10 (4), 553–563. 10.4161/rna.24285 23563448 PMC3710361

[B60] Sobala A. (2013). Small RNAs Derived from the 5′ End of tRNA Can Inhibit Protein Translation in Human Cells. RNA Biol. 10, 553–563. 10.4161/rna.24285 23563448 PMC3710361

[B61] Suzuki T. (2021). The Expanding World of tRNA Modifications and Their Disease Relevance. Nat. Rev. Mol. Cell Biol. 22 (6), 375–392. 10.1038/s41580-021-00342-0 33658722

[B62] Torres A. G. Batlle E. Ribas de Pouplana L. (2014). Role of tRNA Modifications in Human Diseases. Trends Mol. Med. 20 (6), 306–314. 10.1016/j.molmed.2014.01.008 24581449

[B63] Torres A. G. (2019). Enjoy the Silence: Nearly Half of Human tRNA Genes Are Silent. Bioinform Biol. Insights 13, 117793221986845–1177932219868454. 10.1177/1177932219868454 PMC6688141 31447549

[B64] van Raam B. J. Salvesen G. S. (2010). Transferring Death: A Role for tRNA in Apoptosis Regulation. Mol. Cell 37 (5), 591–592. 10.1016/j.molcel.2010.02.001 20227362

[B65] Wang L. Feng X. Jiao Z. Gan J. Meng Q. (2022). Characterization of the Prognostic and Diagnostic Values of ALKBH Family Members in Non-small Cell Lung Cancer. Pathology - Res. Pract. 231, 153809. 10.1016/j.prp.2022.153809 35180653

[B66] Yamasaki S. Ivanov P. Hu G.-f. Anderson P. (2009). Angiogenin Cleaves tRNA and Promotes Stress-Induced Translational Repression. J. Cell Biol. 185 (1), 35–42. 10.1083/jcb.200811106 19332886 PMC2700517

[B67] Yang J. Smith D. K. Ni H. Wu K. Huang D. Pan S. (2020). SOX4-mediated Repression of Specific tRNAs Inhibits Proliferation of Human Glioblastoma Cells. Proc. Natl. Acad. Sci. U.S.A. 117 (11), 5782–5790. 10.1073/pnas.1920200117 32123087 PMC7084149

[B68] Yang W. S. SriRamaratnam R. Welsch M. E. Shimada K. Skouta R. Viswanathan V. S. (2014). Regulation of Ferroptotic Cancer Cell Death by GPX4. Cell 156 (1-2), 317–331. 10.1016/j.cell.2013.12.010 24439385 PMC4076414

[B69] Yi R. Wang H. Deng C. Wang X. Yao L. Niu W. (2020). Dihydroartemisinin Initiates Ferroptosis in Glioblastoma through GPX4 Inhibition. Biosci. Rep. 40 (6). 10.1042/BSR20193314 PMC7313443 32452511

[B70] Ying X. Liu B. Yuan Z. Huang Y. Chen C. Jiang X. (2021). METTL1‐m 7 G‐EGFR/EFEMP1 axis Promotes the Bladder Cancer Development. Clin. Transl. Med 11 (12), e675. 10.1002/ctm2.675 34936728 PMC8694502

[B71] Yu M. Lu B. Zhang J. Ding J. Liu P. Lu Y. (2020). tRNA-Derived RNA Fragments in Cancer: Current Status and Future Perspectives. J. Hematol. Oncol. 13 (1), 121. 10.1186/s13045-020-00955-6 32887641 PMC7487644

[B72] Yu X. Xie Y. Zhang S. Song X. Xiao B. Yan Z. (2021). tRNA-Derived Fragments: Mechanisms Underlying Their Regulation of Gene Expression and Potential Applications as Therapeutic Targets in Cancers and Virus Infections. Theranostics 11 (1), 461–469. 10.7150/thno.51963 33391486 PMC7681095

[B73] Zaborske J. M. Narasimhan J. Jiang L. Wek S. A. Dittmar K. A. Freimoser F. (2009). Genome-wide Analysis of tRNA Charging and Activation of the eIF2 Kinase Gcn2p. J. Biol. Chem. 284 (37), 25254–25267. 10.1074/jbc.M109.000877 19546227 PMC2757228

[B74] Zeng T. Hua Y. Sun C. Zhang Y. Yang F. Yang M. (2020). Relationship between tRNA ‐derived Fragments and Human Cancers. Int. J. Cancer 147 (11), 3007–3018. 10.1002/ijc.33107 32427348

[B75] Zhang J. Jiang H. Shao J. Mao R. Liu J. Ma Y. (2014). SOX4 Inhibits GBM Cell Growth and Induces G0/G1 Cell Cycle Arrest through Akt-P53 axis. BMC Neurol. 14, 207. 10.1186/s12883-014-0207-y 25366337 PMC4233052

[B76] Zhang Y. Qian H. He J. Gao W. (2020). Mechanisms of tRNA-derived Fragments and tRNA Halves in Cancer Treatment Resistance. Biomark. Res. 8, 52. 10.1186/s40364-020-00233-0 33072328 PMC7559774

[B77] Zhang Z. Song X. Feng X. Miao Y. Wang H. Li Y. (2015). Norcantharidin Modulates miR-655-Regulated SENP6 Protein Translation to Suppresses Invasion of Glioblastoma Cells. Tumor Biol. 37, 15635–15641. 10.1007/s13277-015-4447-2 26608369

[B78] Zhao H. Li Y. (2021). Cancer Metabolism and Intervention Therapy. Mol. Biomed. 2 (1), 5. 10.1186/s43556-020-00012-1 35006438 PMC8607959

[B79] Zhao J. Bai H. Li X. Yan J. Zou G. Wang L. (2021). Glucose-sensitive Acetylation of Seryl tRNA Synthetase Regulates Lipid Synthesis in Breast Cancer. Sig Transduct. Target Ther. 6 (1), 303. 10.1038/s41392-021-00714-0 PMC8368063 34400610

[B80] Zhu L. Ge J. Li T. Shen Y. Guo J. (2019). tRNA-derived Fragments and tRNA Halves: The New Players in Cancers. Cancer Lett. 452, 31–37. 10.1016/j.canlet.2019.03.012 30905816

[B81] Zhu L. Li Z. Yu X. Ruan Y. Shen Y. Shao Y. (2021). The tRNA-Derived Fragment 5026a Inhibits the Proliferation of Gastric Cancer Cells by Regulating the PTEN/PI3K/AKT Signaling Pathway. Stem Cell Res. Ther. 12 (1), 418. 10.1186/s13287-021-02497-1 34294122 PMC8296675

